# Whole Body 3.0 T Magnetic Resonance Imaging in Lymphomas: Comparison of Different Sequence Combinations for Staging Hodgkin’s and Diffuse Large B Cell Lymphomas

**DOI:** 10.3390/jpm10040284

**Published:** 2020-12-16

**Authors:** Arash Latifoltojar, Mark K. J. Duncan, Maria Klusmann, Harbir Sidhu, Alan Bainbridge, Deena Neriman, Francesco Fraioli, Jonathan Lambert, Kirit M. Ardeshna, Shonit Punwani

**Affiliations:** 1 Centre for Medical Imaging, University College London, 2nd Floor Charles Bell House, 43-45 Foley Street, London W1W 7TS, UK; a.latifoltojar@nhs.net (A.L.); harbirsidhu@doctors.org.uk (H.S.); 2 The Royal Marsden Hospital, Downs road, Sutton, Surrey SM2 5PT, UK; 3 Department of Radiology, University College London Hospitals NHS Foundation Trust, 235 Euston Road, London NW1 2BU, UK; markduncan2@nhs.net (M.K.J.D.); mklusmann@doctors.org.uk (M.K.); 4 Department of Medical Physics and Biomedical Engineering, University College London Hospitals NHS Foundation Trust, 235 Euston Road, London NW1 2BU, UK; alan.bainbridge@uclh.nhs.uk; 5 Institute of Nuclear Medicine, University College London Hospitals NHS Foundation Trust, 235 Euston Road, London NW1 2BU, UK; deena.neriman@nhs.net (D.N.); fraioli.francesco@gmail.com (F.F.); 6 Department of Haematology, University College London Hospitals NHS Foundation Trust, 235 Euston Road, London NW1 2BU, UK; jonathanlambert@nhs.net (J.L.); kiritardeshna@nhs.net (K.M.A.)

**Keywords:** magnetic resonance imaging, diffusion magnetic resonance imaging, Hodgkin lymphoma, diffuse large-cell lymphoma, cancer staging

## Abstract

To investigate the diagnostic value of different whole-body magnetic resonance imaging (WB-MRI) protocols for staging Hodgkin and diffuse-large B-cell lymphomas (HL and DLBCL), twenty-two patients (M/F 12/10, median age 32, range 22–87, HL/DLBCL 14/8) underwent baseline WB-MRI and ^18^F-2-fluoro-2-deoxy-D-glucose (^18^F-FDG) positron emission tomography (PET) fused with computed tomography (CT) scan ^18^F-FDG-PET-CT. The 3.0 T WB-MRI was performed using pre-contrast modified Dixon (mDixon), T2-weighted turbo-spin-echo (TSE), diffusion-weighted-imaging (DWI), dynamic-contrast-enhanced (DCE) liver/spleen, contrast-enhanced (CE) lung MRI and CE whole-body mDixon. WB-MRI scans were divided into: (1) “WB-MRI *_DWI+IP_*”: whole-body DWI + in-phase mDixon (2) “WB-MRI *_T2-TSE_*”: whole-body T2-TSE (3) “WB-MRI *_Post-C_*”: whole-body CE mDixon + DCE liver/spleen and CE lung mDixon (4) “WB-MRI All “: the entire protocol. Two radiologists evaluated WB-MRIs at random, independently and then in consensus. Two nuclear-medicine-physicians reviewed ^18^F-FDG PET-CT in consensus. An enhanced-reference-standard (ERS) was derived using all available baseline and follow-up imaging. The sensitivity and specificity of WB-MRI protocols for nodal and extra-nodal staging was derived against the ERS. Agreement between the WB-MRI protocols and the ERS for overall staging was assessed using kappa statistic. For consensus WB-MRI, the sensitivity and specificity for nodal staging were 75%, 98% for WB-MRI *_DWI+IP_*, 76%, 98% for WB-MRI *_Post-C_*, 83%, 99% for WB-MRI *_T2-TSE_* and 87%, 100% for WB-MRI *_All_*. The sensitivity and specificity for extra-nodal staging were 67% 100% for WB-MRI *_DWI+IP_*, 89%, 100% for WB-MRI *_Post-C_*, 89%, 100% for WB-MRI *_T2-TSE_* and 100%, 100% for the WB-MRI *_All_*. The consensus WB-MRI *_All_* read had perfect agreement with the ERS for overall staging [kappa = 1.00 (95% CI: 1.00-1.00)]. The best diagnostic performance is achieved combining all available WB-MRI sequences.

## 1. Introduction

Lymphomas, including Hodgkin’s lymphoma (HL) and non-Hodgkin’s lymphoma (NHL), are estimated to account for 3–4% of cancers worldwide [1]. Following histopathological confirmation, accurate staging is of great importance for treatment planning and prognostication. Staging in HL and NHL is predominantly based on the current Lugano classification [2] taking into account the number of involved sites, the type of lesions (nodal or extra-nodal), and the distribution of disease.

The current gold standard imaging for assessment of most common subtypes of adults’ lymphoma is ^18^F-2-fluoro-2-deoxy-D-glucose (^18^F-FDG) positron emission tomography (PET) fused with computed tomography (CT) scan (^18^F-FDG PET-CT) [2,3,4]. However, ^18^F-FDG PET-CT is a cost intensive imaging modality and in many countries access to PET-CT services is geographically limited to large tertiary care centers [5,6]. The more wide-spread availability of magnetic resonance imaging (MRI), coupled with numerous advances in software and hardware developments, makes it a useful technique for studying a range of diseases, including various types of malignancies. Over the past decade, whole-body MRI (WB-MRI) has been developed and investigated as an alternative radiation-free imaging technique and its feasibility has been demonstrated for a range of malignancies including lymphoma [7,8,9,10,11].

However, the most appropriate combination of MRI sequences for use within a WB-MRI protocol remains to be established, and studies to date have used a variety of acquisitions [12,13,14].

Diffusion weighted imaging (DWI) is commonly applied as part of WB-MRI protocols, and may offer an alternative to ^18^F-FDG PET-CT for lymphoma staging [5,13]. Some reports suggest DWI can complement conventional anatomical WB-MRI sequences [14], whilst others indicate that it adds little value to conventional imaging [15].

In this study, we aim to prospectively evaluate the diagnostic performance of differing 3.0 T WB-MRI protocols (comprising combinations of whole body T1 and T2 weighted imaging, DWI and contrast-enhanced (CE) imaging) for initial staging of adult HL and diffuse large B-cell lymphoma (DLBCL, the commonest subtype of NHL) against an enhanced reference standard based on ^18^F-FDG PET-CT and follow-up imaging.

## 2. Materials and Methods

A prospective single-arm observational study was piloted following institutional ethical permission (Research Ethic Committee reference number: 12/LO/0428). Participants were recruited from a single center (blinded for review) and gave written informed consent.

### 2.1. Patient Cohort

Adult patients were identified from a tertiary lymphoma referral center between June 2012 and November 2015 inclusive. Inclusion criteria were: age ≥ 18 years; histopathologically proven HL or DLBCL; no previous malignancy, chemotherapy or radiotherapy; eGFR > 50 mL/min/1.73 m^2^ and no contraindication to MRI.

### 2.2. Study Summary

All recruited patients underwent a multi-parametric (T2 weighted imaging, DWI, T1 and contrast enhanced) WB-MRI scan at baseline in addition to conventional staging imaging (based on ^18^F-FDG PET-CT). Thereafter, as part of usual clinical care, patients underwent interim (where clinically relevant) and end-of-treatment assessments and were then followed for a minimum of 12 months after completion of chemo/radiotherapy. Recruited patients were also asked to attend for subsequent additional multi-parametric WB-MRI scans at the time they were scheduled for interim and/or end-of-treatment conventional imaging assessment.

### 2.3. Multi-Parametric Whole Body MRI Protocol

Imaging was performed using a 3.0 T wide-bore MR scanner (Ingenia; Phillips Healthcare, Best, Netherlands). WB-MRI coverage was from vertex to mid-thigh and was obtained through multi-station acquisition of contiguous body regions with the manufacturers’ head coil, two anterior surface coils and table embedded posterior coils. Full scanning parameters are summarized in Table 1.

In brief, anatomical T1 weighted imaging was performed using a coronal two-point modified Dixon (mDixon) imaging sequence. This was followed by axial T2 weighted turbo spin echo (TSE), axial DWI (with 4 b-values, b0,100,300 and 1000 s/mm^2^) and finally contrast enhanced (CE) MRI acquisitions.

CE MR imaging consisted of axial dynamic contrast enhanced (DCE) MRI of the liver and spleen, coronal CE whole-body mDixon and CE axial mDixon lung MRI. For DCE imaging, pre-contrast mDixon images were acquired in breath-hold to include the entire liver and spleen. This acquisition was then repeated during and after intravenous (IV) injection of 20 mL of gadoterate meglumine (Dotarem, Guebert, France) at 3 mL/s using a pump injector. Multiple arterial, venous and delayed phase acquisitions of liver and spleen were followed by two-stations axial CE lung images from apex of the lung to the top of the liver using mDixon MRI. Finally, whole-body coronal CE mDixon imaging was conducted.

All mDixon images were post-processed online using scanner software to create in-phase, out-of-phase, fat-only and water-only images [16].

### 2.4. ^18^F-FDG PET-CT Protocol

^18^F-FDG PET-CT was performed on a combined GE Discovery LS ^18^F-FDG PET-CT in-line system (GE Healthcare, Milwaukee, Wisconsin, USA). Patients fasted for 6 h and blood glucose levels were tested to exclude hyperglycemia (levels >150 mg/dL).

A standard dose of 5.5 MBq/kg of ^18^F-FDG was intravenously injected 60 min before imaging. Whole-body examinations were performed in the supine position, from skull base to mid-thigh level with 5 bed scans in most of the patients, following the European association of nuclear medicine (EANM) guidelines for injection and scanning [17]. Prior to acquiring the whole-body PET emission scan, a non-contrast CT of the body was obtained using the integrated four-slice CT scanner (140kVp, 80mA tube current, 0.8 s rotation time, 4 × 3.75 mm detectors, pitch 1.5, 5 mm collimation). PET images were reconstructed using the CT for attenuation correction. Combined trans-axial emission images of ^18^F-FDG PET and CT were then reconstructed at 128 × 128 resolution and 2.5 mm thickness.

### 2.5. Whole Body MRI Interpretation

Four WB-MRI datasets were created from each pre-treatment multi-parametric WB-MRI study:

(1) “WB-MRI *_DWI+IP_*”: Whole-body pre-contrast in-phase mDixon + whole-body DWI (b_1000_)
(2) “WB-MRI *_T2-TSE_*”: Whole-body T2-TSE only,
(3) “WB-MRI *_Post-C_*”: Whole-body post-contrast water-only mDixon, DCE liver/spleen and CE lung mDixon,
(4) “WB-MRI *_All_*”: all components from 1–3 above.

Two radiologists (M.K.J.D and M.K with 12 and 6 years of experience) reviewed the anonymized datasets separately, blinded to the clinical history (other than the diagnosis of lymphoma) and all other imaging investigations. All images were reviewed using Osirix (V 4.1, Pixmeo SARL, Bernex, Switzerland) on a Mac (Apple, Cupertino, CA, USA) workstation.

At each reading session, the reporting radiologist evaluated one of each of the four components of the WB-MRI datasets at random. At each reading session, only one dataset for a given patient was revealed to reporting radiologists. To reduce recognition bias, a minimum two-week interval was instituted between reading sessions. A maximum of 6 patients (datasets) were reviewed per session to avoid reader fatigue.

For each dataset, radiologists recorded disease status at 18 nodal sites (cervical [right (R) and left (L)], supraclavicular (R and L), subpectoral (R and L), axillary (R and L), mediastinal, splenic hilar, liver hilar, mesenteric, retroperitoneal, iliac (R and L), inguinal (R and L) and “other” sites, and 12 extra-nodal sites, lung, pleura, pericardium, chest wall, liver, spleen, kidney, stomach, bowel, pancreas, bone and “other” sites) as well as final Lugano classification overall stage (2) were derived.

For the nodal sites, the maximum short-axis dimension of the largest nodal mass in a given region was measured using software calipers. Disease positivity was defined as a mass with a short-axis dimension equal or greater than 1 cm [7,18,19].

The extra-nodal sites were assessed using pre-defined positivity/negativity criteria as described previously [7,18,19,20].

Finally, the time to report each component of WB-MRI read was recorded for each reader.

After completion of the radiologists’ individual reads for all datasets for all patients, a consensus meeting was held between the two radiologists where anatomical sites discrepant for disease positivity for a given patient dataset were re-evaluated to reach a final consensus on disease status. Where no consensus was reached, a third independent radiologist (S.P) was available for adjudication to reach an overall majority opinion.

### 2.6. ^18^F-FDG PET-CT Interpretation

^18^F-FDG PET-CT images were reviewed by two nuclear medicine physicians (F.F and D.N with 10 and 5 years of experience) in consensus. Readers were aware of the diagnosis of lymphoma but were unaware of the WB-MRI findings. All images were assessed on a workstation (Xeleris 2; GE Healthcare, Milwaukee, WI, USA) and results were recorded for the regional divisions defined above for WB-MR imaging.

Nodal dimension was measured on the CT component of the ^18^F-FDG PET-CT and the maximum standardized uptake value (SUV_max_) for the node exhibiting the greatest uptake at each anatomic site was recorded. Disease positivity was defined as the presence of nodes with increased FDG uptake greater than that of the mediastinal and liver pools in a location incompatible with normal physiologic activity [2,21] and/or unexplained nodal enlargement [2]. Extra-nodal disease was defined as previously described [2].

#### Expert Panel Review and Derivation of Enhanced Reference Standard

**Figure 1:**
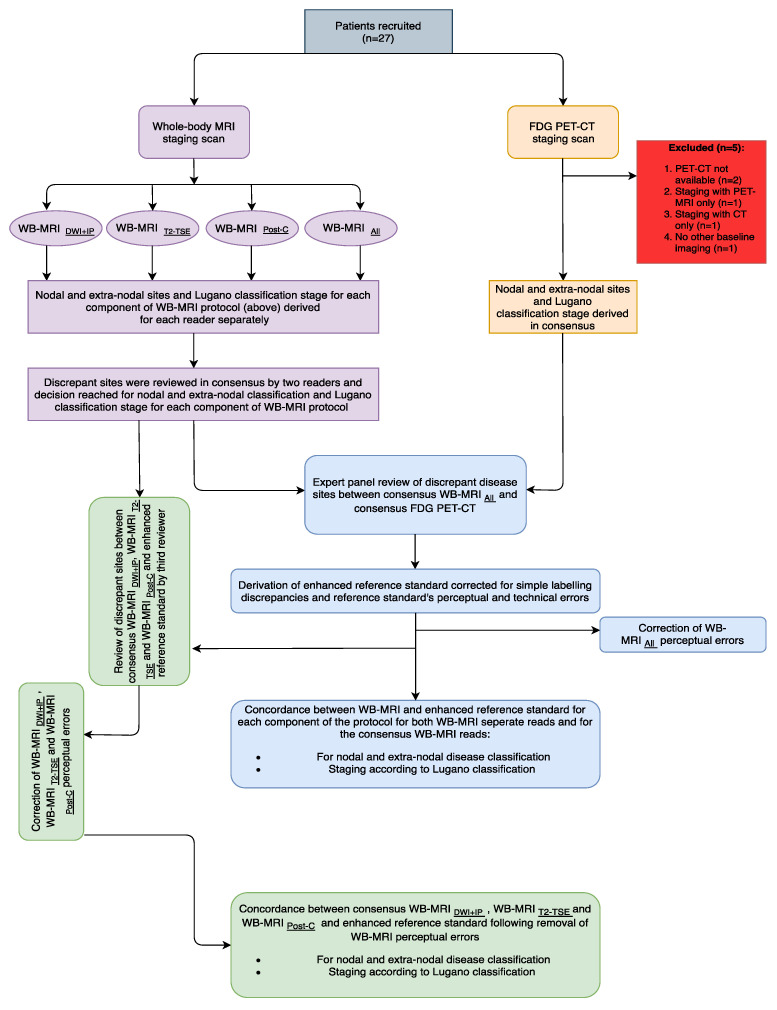
Study flowchart.

Given the potential limitations of standard imaging and the risk of radiologist/nuclear medicine physician perceptual errors [7,20] influencing the WB-MRI and ^18^F-FDG PET-CT staging, a retrospective enhanced reference standard (ERS) was produced to better evaluate the potential accuracy of WB-MRI as previously described [22]. Specifically, all discrepancies between consensus WB-MRI *_All_* and ^18^F-FDG PET-CT at initial staging were reviewed by an expert panel comprising of two reporting radiologists and two reporting nuclear medicine physicians (Figure 1).

The expert panel re-evaluated the ^18^F-FDG PET-CT and the WB-MRI *_All_* dataset. Unlike the initial image interpretations, the panel had access to all clinical data and concurrent imaging investigations, including the follow-up ^18^F-FDG PET-CT and WB-MRI where performed.

Firstly, by directly matching PET-CT and WB-MRI images, the panel corrected for labelling discrepancies resulting from different interpretation of anatomical boundaries between WB-MRI and ^18^F-FDG PET-CT readers [20]. Secondly, based on all the available imaging and follow up data, remaining discrepancies between consensus WB-MRI *_All_* and ^18^F-FDG PET-CT were reviewed to identify and correct for perceptual errors on ^18^F-FDG PET-CT [20]. For example, unequivocal areas of disease positivity on consensus WB-MRI *_All_* that were missed on the original ^18^F-FDG PET-CT interpretation but visible on the ^18^F-FDG PET-CT in retrospect were corrected.

Sites positive for disease on consensus WB-MRI *_All_* but not visible on ^18^F-FDG PET-CT even in retrospect were reviewed in light of other imaging and follow-up to identify technical failures of ^18^F-FDG PET-CT (10). Only unequivocal disease sites on consensus WB-MRI *_All_* that demonstrated a clear response to treatment were considered technical failures of ^18^F-FDG PET-CT (and deemed these positive by ERS); otherwise such findings were classified as consensus WB-MRI *_All_* false positives [20]. In a similar fashion, the panel also identified any false positive findings on ^18^F-FDG PET-CT (and deemed these negative by ERS).

Consensus WB-MRI *_All_* findings discrepant to the ERS were classified into perceptual errors, when the abnormality was visible in retrospect on the WB-MRI, or technical error when it was not [7,20].

Finally, in order to delineate the errors for consensus WB-MRI *_DWI+IP_*, WB-MRI *_T2-TSE_* and WB-MRI *_Post-C_*, a separate review of each dataset compared to ERS was undertaken by a consultant radiologist (H.S with 6 years of experience in WB-MR imaging) who was not involved with the initial image reviewing of the study. All the discrepant sites between WB-MRI *_DWI+IP_*, WB-MRI *_T2-TSE_* and WB-MRI *_Post-C_* and ERS were reviewed and categorized into anatomical boundaries discrepancies, perceptual and technical errors as previously described [20].

### 2.7. Statistical Analysis

Statistical analysis was performed using Prism software (Prism Version 6.0, GraphPad, San Diego, CA, USA) by the study clinical research fellow (blinded for review).

Initially the analysis was performed for nodal and extra-nodal staging, for each of the four WB-MRI generated datasets for each reader against the ERS. Following the initial analysis, the WB-MRI nodal and extra-nodal staging consensus reads (for each component) were compared against the ERS.

For each analysis, the agreement rate, true positive rate (TPR), false positive rate (FPR) and kappa agreement of WB-MRI for nodal and extra-nodal staging were derived.

Agreement between the WB-MRI reads and the ERS for overall staging was tested using a weighted kappa statistic.

The same analysis of agreement rate, TPR, FPR and kappa agreement as well as agreement for overall staging were repeated following correction for anatomical boundaries discrepancies and WB-MRI’s perceptual errors for each of the four WB-MRI generated datasets.

Finally, a repeated measure analysis of variance (ANOVA) with Tukey’s multiple comparison test was used to assess time to report each sequence for each reader; *p*-values < 0.05 were considered as statistically significant.

## 3. Results

### 3.1. Patient Characteristics

The study flowchart is presented in Figure 1.

Twenty-seven patients were prospectively recruited (M: F 13: 14, median age 43, range 22–87 years). Five patients were excluded from the analysis; 1 had ^18^F-FDG PET-MRI, 1 only had whole-body CT scan, 1 did not initially consent to any imaging with radiation exposure and 2 did not have the ^18^F-FDG PET-CT images available for comparison. The demographics, disease subtype, treatment regimen and overall baseline stage of the final 22 patient study cohort is shown in Table 2.

Staging WB-MRI performed within median 10 days (range 0–44 days) of ^18^F-FDG PET-CT without any complication, and before treatment in all patients.

### 3.2. Expert Panel Review and Enhanced Reference Standard

Across the cohort there were 633 anatomical sites (390 nodal and 243 extra-nodal sites) evaluated by both WB-MRI and ^18^F-FDG PET-CT.

**Figure 2:**
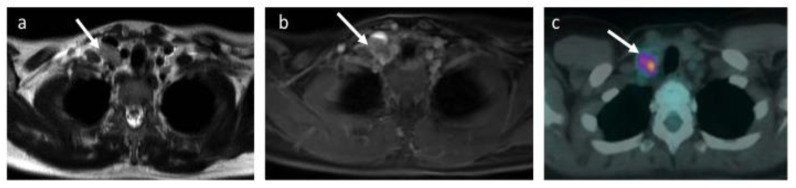
Anatomical boundary descriptive error. Images of a 31-year-old female patient with Ann Arbor stage II Hodgkin’s lymphoma. Patient had bulky mediastinal disease (not shown here) and right-sided supraclavicular nodal involvement. (**a**) T2-TSE, (**b**) contrast-enhanced water-only mDixon and (**c**) fused ^18^F-FDG PET-CT. The right supraclavicular node (arrows) is shown on a, b and c but was considered as supraclavicular on WB-MRI and deep cervical on ^18^F-FDG PET-CT. Following consensus read, the nodal station discrepancy was considered as anatomical boundary descriptive error.

The expert panel consensus review identified and resolved 11 anatomical boundary labelling discrepancies (Figure 2).

**Figure 3:**
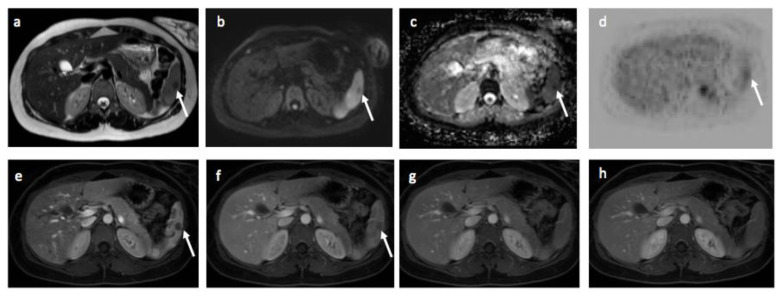
Images of a 24-year-old female patient with Hodgkin’s lymphoma highlighting extra-nodal perceptual error on ^18^F-FDG PET-CT. Splenic involvement was considered positive on WB-MRI and negative on ^18^F-FDG PET-CT. Following the consensus review, the panel corrected the disease site to be positive on ^18^F-FDG PET-CT and subsequently, the enhanced reference standard was positive for splenic involvement. (**a**) T2-TSE, (**b**) DWI b_1000_, (**c**) ADC map, (**d**) ^18^F-FDG PET, (**e**) DCE spleen: early-arterial phase, (**f**) DCE spleen: mid- arterial phase, (**g**,**h**) DCE spleen: late-arterial phase. Arrows showing a focal lesion in spleen.

One ^18^F-FDG PET-CT extra-nodal false negative perceptual error (Figure 3) and one ^18^F-FDG PET-CT nodal false positive perceptual error was identified and corrected, resulting in 52 nodal and 9 extra-nodal positive disease sites on enhanced reference standard.

There were 3 patients with bone marrow (*n* = 3) metastasis as well as spleen (*n* = 1), lung (*n* = 1), pericardium (*n* = 1), chest wall (*n* = 1), liver (*n* = 1) and maxillary sinus (*n* = 1) extra-nodal involvements.

### 3.3. Comparison of Whole-Body MRI and Enhanced Reference Standard

The agreement rate, TPR, FPR and kappa agreement for nodal and extra-nodal staging for each reader and for consensus read of the four generated WB-MRI datasets is summarised in Table 3, Table 4 and Table 5, respectively.

The agreement rate, TPR, FPR and kappa agreement for WB-MRI *_All_* consensus nodal staging were 98%, 87%, 0 and 0.92 (95% CI: 0.86–0.98). The agreement rate, TPR, FPR and kappa agreement for WB-MRI *_All_* consensus extra-nodal staging were 100% 100%, 0 and 1.00 (1.00–1.00).

### 3.4. Comparison of Whole-Body MRI and Enhanced Reference Standard following Correction for Perceptual Errors

**Figure 4:**
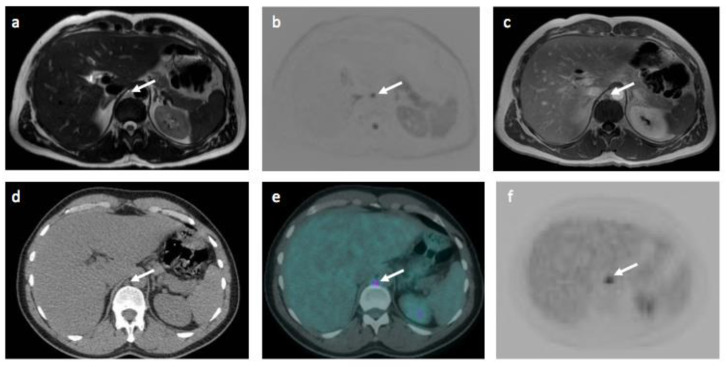
Images of 46-year-old male patient with Hodgkin’s lymphoma highlighting a false negative technical error on WB-MRI. A sub-centimeter FDG avid (SUV_max_ 5.1) retrocrural lymph node that was considered negative nodal site on WB-MRI. The positive nodal station is shown (arrows) on (**a**) T2-TSE, (**b**) DWI b1000 and (**c**) Contrast-enhanced in-phase mDixon WB-MRI images and (**d**) CT scan, (**e**) fused ^18^F-FDG PET-CT and (**f**) ^18^F-FDG PET images.

For consensus WB-MRI *_All_*, there were 7 false negative disease sites due to technical failure in detection of sub-centimeter lymph nodes against the ERS (Figure 4).

Following the additional review by the third radiologist, the anatomical boundaries discrepancies and perceptual errors for consensus WB-MRI *_DWI+IP_* (nodal anatomical boundaries discrepancies: 6, nodal perceptual errors: 2 and extra-nodal perceptual error: 1), consensus WB-MRI *_T2-TSE_* (nodal anatomical boundaries discrepancies: 5, nodal perceptual errors: 1) and consensus WB-MRI *_Post-C_* (nodal anatomical boundaries discrepancies: 6, nodal perceptual errors: 3 and extra-nodal perceptual error: 1) were identified and corrected.

Excluding the 7 false negative nodal disease sites (technical failure in detection of sub-centimeter lymph nodes) on WB-MRI *_All_*, there were additional 3 and 2 technical errors for nodal and extra-nodal sites on WB-MRI *_DWI+IP_* and 1 extra-nodal technical error for WB-MRI *_T2-TSE_* respectively.

The agreement rate, TPR, FPR and kappa agreement for nodal and extra-nodal staging for consensus WB-MRI *_DWI+IP_*, WB-MRI *_T2-TSE_* and WB-MRI *_Post-C_* following correction of the anatomical boundaries discrepancies and perceptual errors are tabulated in Table 6.

### 3.5. Overall Stage

The kappa agreement for the staging based on the Lugano classification (2) of all 4 component of the WB-MRI protocol for each reader and for the consensus read (before and and following correction of the anatomical boundaries discrepancies and WB-MRI perceptual errors) against the ERS is summarised in Table 7.

**Figure 5:**
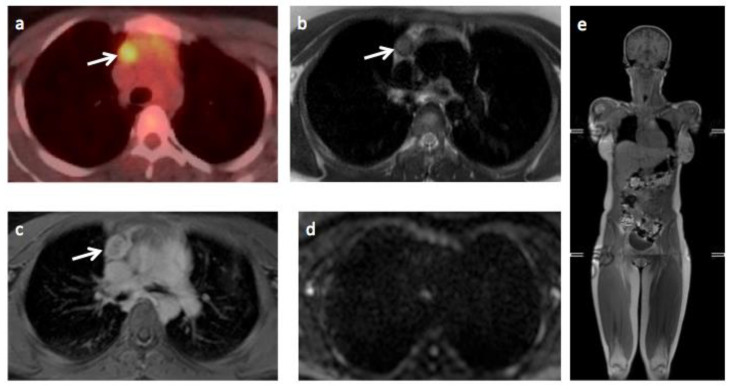
Images of a 22-year-old female patient with stage II Hodgkin’s lymphoma. Patient had left supraclavicular nodal disease (not shown here) and mediastinal disease. (**a**) Fused ^18^F-FDG PET-CT DWI b_1000_ (**b**) T2-TSE and (**c**) contrast-enhanced water-only mDixon showing the mediastinal disease (arrows). Mediastinal disease was not evident in (**d**) DWI b_1000_ and (**e**) pre-contrast in-phase mDixon resulting in under-staging of disease.

Compared to the ERS, WB-MRI *_DWI+IP_* consensus (following correction of the anatomical boundaries discrepancies and perceptual errors) under-staged 3 patients. One patient was under-staged due to false negative interpretation of bone marrow involvement while in the other 2 cases false negative nodal involvement (in mediastinum and supraclavicular stations) resulted in under-staging of disease (Figure 5).

WB-MRI *_T2-TSE_* consensus read under-staged 1 patient due false negative interpretation of bone marrow involvement.

The consensus WB-MRI *_All_* and WB-MRI *_Post-C_* (following correction of the anatomical boundaries discrepancies and perceptual errors) read had perfect agreement with ERS for overall staging according to the Lugano classification (2) [kappa = 1.00 (95% Confidence interval: 1.00-1.00)].

The mean (standard deviation) for time to report WB-MRI *_DWI+IP_*, WB-MRI *_Post-C_*, WB-MRI *_T2-TSE_* and WB-MRI *_All_* were 7.4 (2.4), 7.9 (2.0), 8.5 (2.5) and 8.7 (3.2) minute for reader 1 and 8.1 (2.3), 7.8 (2.4), 8.7 (3.1) and 7.5 (2.3) minute for reader 2, respectively.

For both readers, there was no significant difference for time to report for WB-MRI *_DWI+IP_*, WB-MRI *_Post-C_*, WB-MRI *_T2-TSE_* and WB-MRI *_All_* reads.

## 4. Discussion

In this study we investigated the diagnostic performance of different WB-MRI protocols, using a variety of MRI sequences, as part of a multi-parametric WB-MRI protocol design for staging of HL and DLBCL lymphomas. We found that the overall performance of WB-MRI for nodal and extra-nodal staging was best when all available sequences (WB-MRI *_All_*) were reviewed, both for individual and consensus reads. We also found that for the overall staging, there was a similar pattern of increased agreement with the ERS when all available sequences were assessed concurrently showing a perfect agreement between WB-MRI *_All_* and ERS.

The feasibility of using WB-MRI for staging lymphoma has been investigated in several previous studies [5,7,12,15,20]. Additionally, a more widespread availability of MRI scanners (compare to ^18^F-FDG PET-CT scanners) [6] and lower cost of WB-MRI to ^18^F-FDG PET-CT [23] makes it a potential alternative/adjunct to current gold-standard imaging technique.

For instance, health economy analysis has shown that for staging lung cancers, there is approximately 50% cost reduction for WB-MRI staging compared to standard staging pathway (including PET-CT) [24]. However, the majority of the published work either used a single morphological/functional sequence [22] or investigated the sequential added value of multiple sequences [10,12,15] as part of the WB-MRI protocol. Rarely in the literature has the diagnostic yield of each sequence been investigated separately for the evaluation of the same subject. Kwee et al. [15] reported no additional advantage for supplementing DWI to combined T1 and T2-w WB-MRI for staging lymphomas. In their cohort of 108 patients with various subtypes of lymphoma, they found that T1 and T2-weighted WB-MRI without DWI was concordant with CT staging in 66.6% of cases, compared to 65.4% concordance for that T1 and T2-weighted WB-MRI with DWI.

In our study, WB-MRI *_DWI+IP_* was inferior compared with other sequence combinations for nodal and extra-nodal staging, for both readers and for the consensus read. Following the consensus read and correction of the anatomical boundaries discrepancies and perceptual errors, there were 3 cases that were under-staged with WB-MRI *_DWI+IP_* compared to ERS in our cohort, giving a concordance rate of 86%.

Using a 3.0T WB-MRI, Tsuji et al. [12] showed that whole-body DWI alone was concordant with reference standard ^18^F-FDG PET-CT in 78% (*n* = 22) of 28 patients with DLBCL (*n* = 17) and follicular lymphoma (*n* = 11). However, they also showed that agreement improved (26/28) when T2-weighted imaging was added to whole-body DWI, highlighting the limitation of DWI only imaging for WB-MRI. We observed that WB-MRI *_T2-TSE_* has an improved diagnostic ability compared to WB-MRI *_DWI+IP_* for nodal and extra-nodal disease detection and overall staging. The final consensus WB-MRI *_T2-TSE_* had concordance rate of 95% (21/22) for the overall staging in our cohort, with one patient under-staged due to a false negative interpretation of bone involvement.

We also found that the consensus WB-MRI *_Post-C_* had perfect concordance rate for the overall staging following correction of the anatomical boundaries discrepancies and perceptual errors.

Recently, in a study of 18 patients with various malignancies including lymphoma (*n* = 7), Obara et al. [25] showed that, compared to reference standard ^18^F-FDG PET-CT, CE WB-MRI outperformed both DWI and fat-suppressed T2 only WB-MRI in terms of sensitivity and specificity for malignant disease detection. Whilst our results also suggest a less favourable outcome for WB-MRI *_DWI+IP_* for initial staging of HL and DLBCL, we believe the additional significant information provided by DWI may be helpful for interim and end-of-treatment response evaluation in lymphomas [26,27,28,29].

Of note, we found even following the consensus WB-MRI *_All_* reads, there were seven false negative nodal technical errors, all relating to sub-centimeter FDG avid nodes, corroborating the findings of authors who have highlighted the limitations of size-criteria alone for nodal disease positivity [7]. However, in our cohort the technical errors for WB-MRI *_All_* reads did not change the disease stage of individual patients.

Our study has several limitations. The patient cohort is small and so the power of our study to identify small differences in performance between MRI sequences is statistically limited. We deliberately choose to use size criteria as the primary differentiator of positive and negative nodal status and sequences were used primarily as tools for anatomical localization. Hence, we did not test the value of signal derived quantitative metrics in differentiating between positive and negative nodes. This was informed by growing evidence that application of quantitative metric cut-off values may not be useful in discriminating between positive and negative nodes in lymphoma [20].

Finally, we were unable to obtain histological samples from all suspicious disease sites, as this would not be ethically and/or technically feasible, therefore we used an expert consensus panel and follow-up to derive an enhanced reference standard. Whilst such an approach is imperfect, it is often necessary for diagnostic accuracy studies [7,10,15,20].

## 5. Conclusions

In conclusion, our results suggest that best diagnostic performance is achieved when all imaging sequences are combined (WB-MRI *_All_*). However, this protocol would require 75 min to complete and is unlikely to be suitable for widespread clinical implementation. Where constrained for time, an abbreviated protocol using T2-weighted and contrast-enhanced sequences (which provided best individual performance for nodal and extra-nodal staging respectively) could be considered.

## Figures and Tables

**Table 1:** Whole-body MRI sequence parameters.

	T2-TSE	mDixon (Pre and Post-Contrast ^#^)	DWI (b0, 100, 300, 1000)	DCE (Liver and Spleen)	Post-Contrast Lung
Imaging Plane	Transverse	Coronal	Transverse	Transverse	Transverse
TE (ms)	80	1.02/1.8	71	1.02/1.8	1.02/1.8
TR (ms)	1228	3.0	6371	3.0	3.0
FOV (mm*mm)	500*300	502*300	500*306	512*512	512*512
Voxel Size (mm*mm)	1*1	2.1*2.1	4*4.2	1.2*1.2	1.2*1.2
Number of Slices	40	120	40	80	68
Slice Thickness (mm)	5	5	5	5	6
Acquisition Matrix	500*286	144*238	124*72	256*254	336*332
ETL	91	2	39	2	2
Acceleration Factor (SENSE)	2	2	2.5	2.8	2.8
Pixel Bandwidth (Hz)	537	1992	3369	1890	42055
Acquisition Time per Station (s)	47	17	152	17	17
Number of Stations	6	4	6	1	2

T2-TSE: T2-weighted turbo spin echo, mDixon: modified Dixon, DWI: diffusion weighted imaging, DCE: dynamic contrast enhanced, TE: time of echo, TR: time of repetition, FOV: field of view, ETL: echo train length, SENSE: sensitivity encoding. ^#^ Contrast agent 20 mL intravenous gadoterate meglumine, Dotarem, Guerbet, France.

**Table 2:** Patients’ demographics.

Patient Characteristic (N = 22)	Number or Median (Range)
Age	32 (22–87)
Sex	Male/Female: 12/10
Overall stage	
I	7
II	8
III	2
IV	5
Subtype	
HL	14
DLBCL	8
Chemotherapy regimen	
ABVD	10
RCHOP	8
ABVD-BEACOPP	3
Rituximab	1

HL: Hodgkin’s lymphoma; DLBCL: diffuse large B-cell lymphoma; ABVD: adriamycin, bleomycin, vinblastine, dacarbazine; RCHOP: rituximab, cyclophosphamide, doxorubicin, vincristine, prednisolone; BEACOPP: bleomycin, etoposide, doxorubicin, cyclophosphamide, vincristine, procarbazine, prednisone.

**Table 3:** Comparison of different MRI sequences as part of the WB-MRI protocol for nodal and extra-nodal disease evaluation for reader 1.

Analyses (Reader 1)	Agreement Rate	TPR	FPR	Kappa (95% CI)
**WB-MRI *_DWI+IP_***				
Nodal Sites	92% (360/390)	60% (31/52)	<1% (9/338)	0.63 (0.51–0.75)
Extra-nodal sites	97% (237/243)	44% (4/9)	<1% (1/234)	0.56 (0.25–0.87)
**WB-MRI *_T2-TSE_***				
Nodal Sites	94% (367/390)	66% (34/52)	<1% (5/338)	0.71 (0.60–0.82)
Extra-nodal sites	98% (239/243)	67% (6/9)	<1% (1/234)	0.74 (0.50–0.98)
**WB-MRI *_Post-C_***				
Nodal Sites	96% (373/390)	77% (40/52)	<1% (5/338)	0.80 (0.71–0.89)
Extra-nodal sites	96% (233/243)	67% (6/9)	<1% (7/234)	0.52 (0.27–0.78)
**WB-MRI* _All_***				
Nodal Sites	95% (371/390)	67% (35/52)	<1% (2/338)	0.76 (0.66-0.86)
Extra-nodal sites	>99% (241/243)	89% (8/9)	<1% (1/234)	0.88 (0.73–1.00)

WB-MRI: whole-body magnetic resonance imaging; DWI+IP: whole-body diffusion weighted imaging + pre-contrast in-phase mDixon; Post-C: whole-body post-contrast water only mDixon + dynamic contrast enhanced liver and spleen + contrast enhanced lung; T2-TSE: whole-body T2-weighted turbo spin echo; All: whole-body MRI with all available sequences; TPR: true positive rate; FPR: false positive rate; CI: confidence interval.

**Table 4:** Comparison of different MRI sequences as part of the WB-MRI protocol for nodal and extra-nodal disease evaluation for reader 2.

Analyses (Reader 2)	Agreement Rate	TPR	FPR	Kappa (95% CI)
**WB-MRI *_DWI+IP_***				
Nodal Sites	95% (369/390)	73% (38/52)	<1% (7/338)	0.75 (0.65–0.85)
Extra-nodal sites	97% (237/243)	44% (4/9)	<1% (1/234)	0.56 (0.25–0.87)
**WB-MRI *_T2-TSE_***				
Nodal Sites	96% (374/390)	75% (39/52)	<1% (3/338)	0.81 (0.71–0.90)
Extra-nodal sites	>99% (241/243)	89% (8/9)	<1% (1/234)	0.88 (0.73–1.00)
**WB-MRI *_Post-C_***				
Nodal Sites	96% (374/390)	85% (44/52)	<1% (8/338)	0.82 (0.74–0.91)
Extra-nodal sites	99% (240/243)	78% (7/9)	<1% (7/234)	0.59 (0.35–0.83)
**WB-MRI* _All_***				
Nodal Sites	97% (378/390)	83% (43/52)	<1% (3/338)	0.86 (0.78 to 0.94)
Extra-nodal sites	>99% (242/243)	89% (8/9)	0% (0/234)	0.94 (0.82 to 1.00)

WB-MRI: whole-body magnetic resonance imaging; DWI+IP: whole-body diffusion weighted imaging + pre-contrast in-phase mDixon; Post-C: whole-body post-contrast water only mDixon + dynamic contrast enhanced liver and spleen + contrast enhanced lung; T2-TSE: whole-body T2-weighted turbo spin echo; All: whole-body MRI with all available sequences; TPR: true positive rate; FPR: false positive rate; CI: confidence interval.

**Table 5:** Comparison of different MRI sequences as part of the WB-MRI protocol for nodal and extra-nodal disease evaluation for the consensus read.

Analyses (Consensus)	Agreement Rate	TPR	FPR	Kappa (95% CI)
**WB-MRI *_DWI+IP_***				
Nodal Sites	95% (372/390)	75% (39/52)	<1% (5/338)	0.79 (0.69–0.88)
Extra-nodal sites	97% (240/243)	67% (6/9)	0% (0/234)	0.79 (0.57–1.00)
**WB-MRI *_T2-TSE_***				
Nodal Sites	97% (377/390)	83% (43/52)	<1% (4/338)	0.85 (0.77–0.93)
Extra-nodal sites	>99% (242/243)	89% (8/9)	0% (0/234)	0.93 (0.82–1.00)
**WB-MRI *_Post-C_***				
Nodal Sites	96% (373/390)	77% (40/52)	<1% (5/338)	0.80 (0.71–0.89)
Extra-nodal sites	>99% (242/243)	89% (8/9)	0% (0/234)	0.94 (0.82–1.00)
**WB-MRI* _All_***				
Nodal Sites	98% (383/390)	87% (45/52)	0% (0/338)	0.92 (0.86–0.98)
Extra-nodal sites	100% (243/243)	100% (9/9)	0% (0/234)	1.00 (1.00–1.00)

WB-MRI: whole-body magnetic resonance imaging; DWI+IP: whole-body diffusion weighted imaging + pre-contrast in-phase mDixon; Post-C: whole-body post-contrast water only mDixon + dynamic contrast enhanced liver and spleen + contrast enhanced lung; T2-TSE: whole-body T2-weighted turbo spin echo; All: whole-body MRI with all available sequences; TPR: true positive rate; FPR: false positive rate; CI: confidence interval.

**Table 6:** Comparison of different MRI sequences as part of the WB-MRI protocol for nodal and extra-nodal disease evaluation for the consensus read following correction of the anatomical boundaries discrepancies and WB-MRI perceptual errors.

Analyses (Consensus/Post Correction)	Agreement Rate	TPR	FPR	Kappa (95% CI)
**WB-MRI *_DWI+IP_***				
Nodal Sites	97% (380/390)	81% (42/52)	0% (0/338)	0.88 (0.81–0.95)
Extra-nodal sites	>99% (241/243)	78% (7/9)	0% (0/234)	0.87 (0.69–1.00)
**WB-MRI *_T2-TSE_***				
Nodal Sites	98% (383/390)	87% (45/52)	0% (0/338)	0.92 (0.86–0.98)
Extra-nodal sites	>99% (242/243)	89% (8/9)	0% (0/234)	0.93 (0.82–1.00)
**WB-MRI *_Post-C_***				
Nodal Sites	98% (383/390)	87% (45/52)	0% (0/338)	0.92 (0.86–0.98)
Extra-nodal sites	100% (243/243)	100% (9/9)	0% (0/234)	0.94 (0.84–0.97)
**WB-MRI* _All_***				
Nodal Sites	98% (383/390)	87% (45/52)	0% (0/338)	0.92 (0.86–0.98)
Extra-nodal sites	100% (243/243)	100% (9/9)	0% (0/234)	1.00 (1.00–1.00)

WB-MRI: whole-body magnetic resonance imaging; DWI+IP: whole-body diffusion weighted imaging + pre-contrast in-phase mDixon; Post-C: whole-body post-contrast water only mDixon + dynamic contrast enhanced liver and spleen + contrast enhanced lung; T2-TSE: whole-body T2-weighted turbo spin echo; All: whole-body MRI with all available sequences; TPR: true positive rate; FPR: false positive rate; CI: confidence interval.

**Table 7:** Evaluation of different MRI sequences as part of the WB-MRI protocol for overall staging compared to enhanced reference standard staging. Weighted kappa agreement and 95% confidence interval are shown.

	WB-MRI			
	**DWI+IP**	**Post-C**	**T2-TSE**	**All**
Reader 1	0.55 (0.30–0.80)	0.69 (0.46–0.93)	0.69 (0.46–0.91)	0.88 (0.72–0.97)
Reader 2	0.62 (0.37–0.87)	0.76 (0.53–0.98)	0.88 (0.71–1.00)	0.88 (0.71–1.00)
Consensus	0.75 (0.52–0.97)	0.94 (0.82–1.00)	0.94 (0.82–1.00)	1.00 (1.00–1.00)
Post-correction	0.81 (0.61–1.00)	1.00 (1.00–1.00)	0.94 (0.82–1.00)	1.00 (1.00–1.00)

WB-MRI: whole-body magnetic resonance imaging; DWI+IP: whole-body diffusion weighted imaging + pre-contrast in-phase mDixon; Post-C: whole-body post-contrast water only mDixon + dynamic contrast enhanced liver and spleen + contrast enhanced lung; T2-TSE: whole-body T2-weighted turbo spin echo; All: whole-body MRI with all available sequences; 95% CI: 95% confidence interval
